# Performance of the Finnish Diabetes Risk Score and a Simplified Finnish Diabetes Risk Score in a Community-Based, Cross-Sectional Programme for Screening of Undiagnosed Type 2 Diabetes Mellitus and Dysglycaemia in Madrid, Spain: The SPREDIA-2 Study

**DOI:** 10.1371/journal.pone.0158489

**Published:** 2016-07-21

**Authors:** M. A. Salinero-Fort, C. Burgos-Lunar, C. Lahoz, J. M. Mostaza, J. C. Abánades-Herranz, F. Laguna-Cuesta, E. Estirado-de Cabo, F. García-Iglesias, T. González-Alegre, B. Fernández-Puntero, L. Montesano-Sánchez, D. Vicent-López, V. Cornejo-del Río, P. J. Fernández-García, V. Sánchez-Arroyo, C. Sabín-Rodríguez, S. López-López, P. Patrón-Barandio, P. Gómez-Campelo, SPREDIA-2 Group

**Affiliations:** 1 Subdirección General de Investigación Sanitaria, Consejería de Sanidad de Madrid, Madrid, Spain; 2 MADIABETES Research Group. Madrid, Spain; 3 Aging and Fragility in the Elderly Group- IdiPAZ, Madrid, Spain; 4 Red de Investigación en Servicios de Salud en Enfermedades Crónicas (REDISSEC), Madrid, Spain; 5 Dirección General de Salud Pública, Subdirección de Promoción, Prevención y Educación de la Salud, Consejería de Sanidad, Madrid, Spain; 6 Servicio de Medicina Interna, Hospital Carlos III, Madrid, Spain; 7 Centro de Salud Monóvar, Servicio Madrileño de Salud, Madrid, Spain; 8 Hospital Carlos III, Madrid, Spain; 9 Hospital de Fuenlabrada, Madrid, Spain; 10 Plataforma de Apoyo al Investigador Novel (PAIN Platform), Hospital La Paz Institute for Health Research (IdiPAZ), Madrid, Spain; University of Catanzaro, ITALY

## Abstract

**Aim:**

To evaluate the performance of the Finnish Diabetes Risk Score (FINDRISC) and a simplified FINDRISC score (MADRISC) in screening for undiagnosed type 2 diabetes mellitus (UT2DM) and dysglycaemia.

**Methods:**

A population-based, cross-sectional, descriptive study was carried out with participants with UT2DM, ranged between 45–74 years and lived in two districts in the north of metropolitan Madrid (Spain). The FINDRISC and MADRISC scores were evaluated using the area under the receiver operating characteristic curve method (ROC-AUC). Four different gold standards were used for UT2DM and any dysglycaemia, as follows: fasting plasma glucose (FPG), oral glucose tolerance test (OGTT), HbA1c, and OGTT or HbA1c. Dysglycaemia and UT2DM were defined according to American Diabetes Association criteria.

**Results:**

The study population comprised 1,426 participants (832 females and 594 males) with a mean age of 62 years (SD = 6.1). When HbA1c or OGTT criteria were used, the prevalence of UT2DM was 7.4% (10.4% in men and 5.2% in women; p<0.01) and the FINDRISC ROC-AUC for UT2DM was 0.72 (95% CI, 0.69–0.74). The optimal cut-off point was ≥13 (sensitivity = 63.8%, specificity = 65.1%). The ROC-AUC of MADRISC was 0.76 (95% CI, 0.72–0.81) with ≥13 as the optimal cut-off point (sensitivity = 84.8%, specificity = 54.6%). FINDRISC score ≥12 for detecting any dysglycaemia offered the best cut-off point when HbA1c alone or OGTT and HbA1c were the criteria used.

**Conclusions:**

FINDRISC proved to be a useful instrument in screening for dysglycaemia and UT2DM. In the screening of UT2DM, the simplified MADRISC performed as well as FINDRISC.

## Introduction

Diabetes mellitus (DM) affects around 8.3% of the adult population worldwide, and the total number of cases is predicted to rise from 371 million in 2012 to 552 million in 2030 [1]. This increase may be due to the rising prevalence of overweight and obesity, a generalized decrease in physical activity, and changes in the demographic structure of the population [2].

Over 90% of patients with DM have type 2 diabetes mellitus (T2DM), and over 50% of cases are undiagnosed [1]. Patients with T2DM can remain asymptomatic for a long time with elevated blood glucose levels, blood pressure, and cholesterol. In fact, diagnosis is often not confirmed until the development of serious complications, whose management is more difficult and expensive.

Consequently, interest in identifying individuals with undiagnosed T2DM (UT2DM) is high, considering that there is strong evidence that the progression of uncomplicated T2DM to complicated T2DM can be slowed or stopped with lifestyle modifications [3] or pharmacological interventions [4].

When patients are diagnosed with DM or impaired glucose tolerance, they have frequently already developed subclinical atherosclerosis [5]. Therefore, early diagnosis of DM could favor the implementation of measures aimed at preventing cardiovascular complications.

The prevalence of DM is lower in countries where the Mediterranean diet is followed [6]. Consequently, it is necessary to evaluate the diagnostic performance of diabetes screening tools in these countries, since variations in prevalence lead to considerable changes in the predictive values of the tools.

Furthermore, findings in the literature suggest that every year around 5%-10% of individuals with prediabetes, ie, those with impaired fasting glucose and impaired glucose tolerance, have a high risk of being diagnosed with T2DM [7]. It seems that earlier detection of this population and follow-up with intervention strategies could delay or prevent T2DM, improve glycemic control, and even decrease the incidence of DM and the development of associated complications [8].

DM risk scores [9] constitute an easy, non-invasive, and inexpensive approach to the assessment of an individual’s risk of UT2DM and dysglycaemia that can reduce the number of people who have to undergo diagnostic glucose tolerance tests [10].

A recent systematic review of 17 risk scores used to detect UT2DM [11] showed that the vast majority of these scores include the classic risk factors for T2DM: age, sex, body mass index (BMI), family history of DM, blood pressure medication, physical activity, waist circumference, and diet.

The Finnish Diabetes Risk Score (FINDRISC) is one of the most frequently used instruments for assessing the risk of DM [12]. It comprises only eight variables associated with anthropometric parameters and lifestyle factors: age, BMI, waist circumference, family history of diabetes, use of blood pressure medication, history of elevated blood glucose, daily physical activity, and daily consumption of vegetables, fruit, and berries. FINDRISC assesses whether an individual has UT2DM or dysglycaemia or the probability of developing T2DM during the following 10 years.

DM risk scores must be calibrated so that they can be applied in different populations and countries. In Spain, only two studies [13,14] have validated the ability of the FINDRISC score for detection of UT2DM. The cut-off points proposed by these studies (≥15 and ≥9) differ from those of the original FINDRISC, probably owing to differences between the populations (age, risk of cardiovascular disease). However, to our knowledge, no studies have examined the population at risk of UT2DM measured using the FINDRISC in Spain.

Given that time is a limited resource in the primary care setting [15], simplifying the FINDRISC score may improve its efficiency.

We performed a cross-sectional study to evaluate the performance of FINDRISC and a simplified version of FINDRISC—MADRISC—for screening of UT2DM and any dysglycaemia in a representative sample of the Spanish population living in Madrid.

## Material and Methods

### Design

This study was conducted as part of a broader project, the **S**creening **PRE**-diabetes and type 2 **DIA**betes (SPREDIA-2) study, which has been described in detail elsewhere [16]. SPREDIA-2 is a population-based prospective cohort study in which baseline screening was performed from July 2010 to March 2014 according to Standards for Reporting of Diagnostic Accuracy criteria [17].

### Subjects

A total of 2,553 subjects were contacted. Potential participants were selected randomly from the electronic health records of all patients with health care coverage from two districts in the north metropolitan area of Madrid (Spain), namely, Fuencarral-El Pardo and Tetuán, which include three and seven primary health care centers, respectively. Of the 1,592 subjects (62.4%) who agreed to participate, 1,426 had not been diagnosed with DM.

Those subjects not interested in participating were asked to report voluntary sociodemographic and clinical data, which revealed no significant differences in age, sex, or BMI, except for a history of high blood pressure, dyslipidemia and family history of DM (S1 Table).

The study procedure has been described in detail elsewhere (16). Briefly, recruitment was divided into three phases. First, the potential participants were sent a letter signed by their general practitioner explaining the objectives of the study and inviting them to participate. Second, subjects were contacted by phone to resolve doubts, and, if they were interested in participating, were given an appointment for the assessment. To minimize the losses attributable to failure to locate the patient, up to four telephone calls were made at different times and on different days. Third, the patient attended the assessment in the outpatient clinic of Carlos III Hospital after an overnight fast. Upon arrival, a fasting blood analysis was obtained by measuring blood levels of glucose, creatinine, uric acid, HbA1c, serum insulin, and lipids and lipoproteins. Immediately after blood sampling, all subjects with no previous diagnosis of diabetes underwent an oral glucose tolerance test (OGTT) with 75 g of anhydrous glucose in a total fluid volume of 300 ml. A second blood sample was obtained 2 hours later.

### Variables

#### FINDRISC

All individuals completed the FINDRISC [12], an 8-item score (0–26 points) on which a higher score indicates a high risk of T2DM.

Body weight and height were measured in light clothing and without shoes, and waist circumference was measured midway between the lowest rib and the iliac crest.

#### Laboratory measurements

All participants with no previous diagnosis of DM at baseline underwent a standard OGTT, which was carried out according to World Health Organization (WHO) recommendations [18], and in which FPG and glucose (OGTT) were measured (FPG using the glucose oxidase method). HbA1c was measured using high-performance liquid chromatography, based on the National Glycohemoglobin Standardization Program standardized to the Diabetes Control and Complications Trial [19]. Total cholesterol and triglyceride levels were measured using the standard enzymatic automated method. Low-density lipoprotein (LDL) cholesterol was calculated in subjects with triglycerides <400 mg/dL using the Friedewald formula, as follows: [LDL cholesterol = total cholesterol–(high-density lipoprotein [HDL] cholesterol + triglyceride/5)]. HDL cholesterol was measured after precipitation of apo-B lipoproteins. Serum insulin was determined by immunoassay using an Immulite 2000 analyzer (Siemens Healthcare Diagnostics, Erlangen, Germany).

We also recorded sociodemographic variables (age, educational level), clinical variables (family history of DM, family history of high blood pressure, smoking), and prescribed treatments.

The study protocol was designed in advance, and the interviewers received homogeneous training in the evaluation procedure of the study to minimize variability in data collection. The FINDRISC questionnaire was self-administered. Clinical variables and prescribed treatments were recorded by physicians and anthropometric parameters by nurses.

### Definitions

Participants were classified using current American Diabetes Association criteria [20]. In healthly participants (no previous diagnosis of DM), unknown impaired glucose metabolism, also referred to as prediabetes, was defined as FPG ≥100–125 mg/dl, or HbA1c of 5.7–6.4%, or an OGTT result of 140–199 mg/dl. Participants with no previous diagnosis of DM and FPG ≥126 mg/dl or HbA1c levels ≥6.5% or an OGTT result of ≥200 mg/dl were considered to have UDM. Dysglycaemia was defined as the presence of prediabetes or DM.

Four different gold standards were used for the diagnosis of T2DM and any dysglycaemia, as follows: FPG, OGTT, HbA1c, and OGTT or HbA1c.

### Ethics statement

The Ethics Committee of the Carlos III Hospital (Madrid, Spain) approved the study, and the participants gave their written informed consent.

### Statistical methods

Statistical methods included the *t* test, one-way analysis of variance, and the chi-square test. To assess the performance of the FINDRISC questionnaire in UT2DM and dysglycaemia, receiver operating characteristic (ROC) curves were plotted, and sensitivity, specificity, positive predictive value, negative predictive value, and positive and negative likelihood ratios were calculated. The 95% confidence intervals (95% CI) were calculated using exact methods. The optimal cut-off points used were the peaks of the ROC curve, where the sum of sensitivity and specificity was at a maximum.

To develop the MADRISC, we first estimated the score of each variable on the FINDRISC using univariate logistic regression. We then selected the three variables most strongly associated with UDM to be included in the multivariate logistic regression model. Third, in order to derive the scores allocated to each variable of the new score, the beta coefficients obtained in the multivariate model were multiplied by 10. The total score of the MADRISC was the sum of these coefficients, which ranged from 0 to 41. Higher scores correspond to an increasing risk of DM.

The statistical analysis was performed using SPSS Statistics for Windows, version 21.0 (IBM Corp, Armonk, New York, USA) and MedCalc for Windows, version 15.8 (MedCalc Software bvba, Ostend, Belgium).

## Results

The baseline characteristics of the 1,426 participants who did not have known DM are shown in Table 1. Participants were aged 61.7 years (SD = 6), and 58.3% (n = 832) were women. One-third of the participants had a family history of diabetes (31.6%), were hypertensive (32.6%), and met the criteria for metabolic syndrome (32.5%). Males had a significantly greater disease burden (hypertension, coronary artery disease, peripheral artery disease, and metabolic syndrome) and more frequently received cardiovascular drugs (aspirin and renin-angiotensin system blockers). Furthermore, males had higher values for blood pressure, triglycerides, uric acid, and serum insulin. There were no differences between males and females for the FINDRISC score.

**Table 1:** Characteristics of the study participants.

		Free DM by gender (n = 1,426)		
	Total	Men	Women	p-value
**Subjects,** % (n)	100 (1,426)	41.7 (594)	58.3 (832)	
**Age**, *mean (SD)*	61.7 (6)	61.4 (6.2)	62 (5.9)	0.14
**University studies**, % (n)	33 (472)	44.3 (263)	25.1 (209)	<0.01
**Current smoking**, % (n)	16.5 (235)	19.4 (115)	14.4 (120)	<0.01
**Waist circumference**, cm *mean (SD)*	94.5 (12.2)	101 (10.1)	90 (11.6)	<0.01
**Family history of DM**, % (n)	31.6 (450)	28.3 (168)	33.9 (282)	0.03
**Family history of high blood pressure**, % (n)	48.7 (694)	39.4 (234)	55.3 (460)	<0.01
**Hypertension,** % (n)	32.6 (465)	36.9 (219)	29.6 (246)	<0.01
**Treated hypertension,** % (n)	29.2 (416)	33.3 (198)	26.2 (218)	0.66
**Coronary artery disease,** % (n)	2.9 (42)	5.9 (35)	0.8 (7)	<0.01
**Peripheral artery disease,** % (n)	0.9 (13)	1.9 (11)	0.2 (2)	<0.01
**Stroke,** % (n)	2.1 (30)	2.7 (16)	1.7 (14)	0.19
**Chronic atrial fibrillation,** % (n)	2.2 (32)	3 (18)	1.7 (14)	0.09
**Dyslipidemia,** % (n)	45.4 (647)	45.1 (268)	45.6 (379)	0.89
**Treated dyslipidemia,** % (n)	24.8 (353)	23.1 (137)	26 (216)	0.09
**Metabolic syndrome,** % (n)	32.5 (464)	38.4 (228)	28.4 (236)	<0.01
**Renin-angiotensin system blockers,** % (n)	22.1 (315)	28.5 (169)	17.5 (146)	<0.01
**Statins,** % (n)	24.1 (343)	24.2 (144)	23.9 (199)	0.89
**Aspirin,** % (n)	6 (86)	9.1 (54)	3.8 (32)	<0.01
**BMI**, kg/m^2^ *mean (SD)*	28.2 (4.7)	28.7 (4)	27.9 (5.1)	<0.01
**Plasma glucose 0 h**, mg/dl *mean (SD)*	102 (12.4)	106 (13.9)	99 (10.4)	<0.01
**Plasma glucose 2 h**, mg/dl *mean (SD)*	122 (42.9)	131 (48.5)	116 (37.1)	<0.01
**HbA1c <6.5%**	96.8 (1,380)	95.6 (568)	97.6 (812)	0.03
**HbA1c,** % *mean (SD)*	5.7 (0.4)	5.7 (0.4)	5.7 (0.3)	0.20
**Systolic blood pressure,** mmHg, mean (SD)	124 (16.9)	128 (15.8)	121 (17.1)	<0.01
**Diastolic blood pressure,** mmHg, mean (SD)	77 (9.9)	79 (9.9)	75.8 (9.7)	<0.01
**Total cholesterol,** mg/dl, mean (SD)	211 (36.2)	202 (36.6)	217 (34.6)	<0.01
**HDL cholesterol,** mg/dl, mean (SD)	55 (14.6)	47 (11.3)	60 (14.4)	<0.01
**LDL cholesterol,** mg/dl, mean (SD)	136 (32)	132 (32.7)	139 (31.2)	<0.01
**Triglycerides**, mg/dl, *mean (SD)*	102 (69.5)	114 (69.5)	93 (68.2)	<0.01
**Serum insulin 0 h,** mU/l, *mean (SD)*	10.3 (8.5)	11.6 (8.7)	9.4 (8.3)	<0.01
**Uric acid,** mg/dl, *mean (SD)*	5.3 (1.3)	6 (1.2)	4.7 (1.1)	<0.01
**FINDRISC score**, *mean (SD)*	11.3 (4.4)	11.2 (4.3)	11.4 (4.4)	0.20

The prevalence of UDM and dysglycaemia varied greatly depending on the criteria used (Table 2). Based on OGTT or HbA1c, the prevalence of UDM was 7.4% (95% CI, 6.0–8.8%), and the prevalence of dysglycaemia was 59.7% (95% CI, 57.2–62.3%). Based on OGTT criteria alone, the prevalence of UDM fell to 5.6% (95% CI, 4.4–6.8%) and the prevalence of dysglycaemia to 24.9% (95% CI, 22.6–27.2%). Based on HbA1c criteria alone, the prevalence of UDM was 3.2% (95% CI, 2.3–4.1%) and the prevalence of dysglycaemia was 51.6% (95% CI, 49.0–54.2%). The prevalence of UDM was significantly higher in males than in females (p<0.05), regardless of the criteria used.

**Table 2:** Prevalence of undiagnosed diabetes mellitus and dysglycaemia. UDM: undiagnosed diabetes mellitus; FPG: fasting plasm glucose; OGTT: oral glucose tolerance test; HbA1c: glycated haemoglobin.

	Diagnostic Criterion (n)	Prevalence %			p value
		Overall (95% CI)	Male (n/N)	Female (n/N)	
**UDM**					
	FPG (1,426)	4.3 (3.3–5.4)	7.6 (45/594)	2.0 (17/832)	<0.01
	OGTT (1,408)	5.6 (4.4–6.8)	8.3 (49/588)	3.8 (31/820)	<0.01
	HbA1c (1,425)	3.2 (2.3–4.1)	4.4 (26/594)	2.3 (19/831)	0.03
	OGTT and/or HbA1c (1,426)	7.4 (6.0–8.8)	10.4 (62/594)	5.2 (43/832)	<0.01
**Dysglycaemia**					
	FPG (1,426)	48.5 (45.9–51.1)	59.3 (352/594)	40.9 (340/832)	<0.01
	OGTT (1,408)	24.9 (22.6–27.2)	31.8 (187/588)	19.9 (163/820)	<0.01
	HbA1c (1,425)	51.6 (49.0–54.2)	47.6 (283/594)	54.5 (453/831)	0.01
	OGTT and/or HbA1c (1,426)	59.7 (57.2–62.3)	58.8 (349/594)	60.3 (502/832)	0.55

Data on the performance of the FINDRISC score to identify UT2DM according to OGTT or HbA1c showed that the optimal cut-off point was ≥13, at which sensitivity was 63.8% and specificity 65.1%; for a cut-off value of ≥9, sensitivity was 96.2% and specificity 29.8% (Table 3).

**Table 3:** Characteristics of the FINDRISC questionnaire using different cut-off values for screen-detected unknown type 2 diabetes or newly diagnosed type 2 diabetes.

GOLD STANDARD	FINDRISC Cut-off	Sensitivity (%) (95%CI)	Specificity (%) (95%CI)	PPV (%) (95%CI)	NPV (%) (95%CI)	LR+ (95%CI)	LR-(95%CI)	N (%)
HbA1c (N: 1,425 participants)								
	≥9	100.0 (90.2–99.8)	28.8 (26.4–31.3)	4.4 (3.3–5.9)	99.9 (98.8–100)	1.40 (1.36–1.45)		1,028 (72.1)
	≥10	86.7 (72.5–94.5)	37.0 (34.5–39.7)	4.4 (3.1–5.9)	98.8 (97.4–99.5)	1.38 (1.22–1.55)	0.36 (0.17–0.76)	908 (63.7)
	≥11	82.2 (67.4–91.5)	46.2 (43.5–48.8)	4.7 (3.4–6.5)	98.8 (97.5–99.4)	1.53 (1.32–1.76)	0.39 (0.20–0.72)	780 (54.7)
	≥12	77.8 (62.5–88.3)	55.9 (53.2–58.5)	5.4 (3.9–7.6)	98.7 (97.6–99.3)	1.76 (1.49–2.08)	0.40 (0.23–0.69)	644 (45.2)
	≥13	71.1 (55.5–83.2)	64.1 (61.5–66.6)	6.1 (4.3–8.5)	98.6 (97.5–99.2)	1.98 (1.62–2.41)	0.45 (0.28–0.71)	528 (37.0)
	**≥14**	**64.4 (48.7–77.7)**	**73.4 (71–75.7)**	**7.3 (5.0–10.5)**	**98.5 (97.4–99.1)**	**2.42 (1.92–3.06)**	**0.48 (0.33–0.72)**	**396 (27.8)**
	≥15	51.1 (36.0–66.1)	79.1 (76.9–81.2)	7.4 (4.9–11.0)	98.0 (97.0–98.7)	2.45 (1.81–3.32)	0.62 (0.46–0.83)	311 (21.8)
	≥16	44.4 (30.0–59.9)	85.7 (83.7–87.5)	9.2 (5.9–14.1)	97.9 (96.9–98.6)	3.11 (2.19–4.42)	0.65 (0.50–0.84)	217 (15.2)
OGTT (N: 1,408 participants)								
	≥9	95.0 (87.0–98.4)	29.4 (27–32)	7.5 (6–9.3)	99.0 (97.3–99.7)	1.35 (1.27–1.43)	0.17 (0.07–0.44)	1,028 (72.1)
	≥10	83.8 (73.5–90.7)	37.6 (35–40.3)	7.5 (5.9–9.5)	97.5 (95.6–98.6)	1.34 (1.21–1.49)	0.43 (0.26–0.71)	908 (63.7)
	≥11	76.3 (65.2–84.8)	46.6 (43.9–49.3)	7.9 (6.2–10.1)	97 (95.3–98.2)	1.43 (1.25–1.63)	0.51 (0.34–0.76)	780 (54.7)
	≥12	65.0 (53.4–75.1)	56.0 (53.3–58.7)	8.2 (6.2–10.7)	96.4 (94.7–97.5)	1.48 (1.24–1.76)	0.62 (0.46–0.85)	644 (45.2)
	≥13	58.8 (47.2–69.5)	64.3 (61.7–66.9)	9.0 (6.8–11.9)	96.3 (94.8–97.4)	1.65 (1.35–2.01)	0.64 (0.49–0.84)	528 (37.0)
	≥14	48.8 (37.5–60.1)	73.5 (71–75.8)	10.0 (7.3–13.5)	96.0 (94.5–97.1)	1.84 (1.44–2.34)	0.70 (0.56–0.87)	396 (27.8)
	**≥15**	**45 (40.0–56.5)**	**79.5 (77.2–81.6)**	**11.7 (8.4–15.9)**	**96.0 (94.6–97.0)**	**2.20 (1.69–2.86)**	**0.69 (0.57–0.84)**	**311 (21.8)**
	≥16	35 (24.9–46.6)	85.9 (83.9–87.7)	13.0 (9.0–18.4)	95.6 (94.3–96.7)	2.49 (1.79–3.45)	0.76 (0.64–0.89)	217 (15.2)
FPG (N: 1,426 participants)								
	≥9	93.6 (83.5–97.9)	28.9 (26.5–31.4)	5.6 (4.4–7.3)	99.0 (97.3–99.7)	1.32 (1.22–1.42)	0.22 (0.09–0.58)	1,028 (72.1)
	≥10	85.5 (73.7–92.8)	37.3 (34.8–40.0)	5.8 (4.4–7.6)	98.3 (96.6–99.2)	1.36 (1.22–1.52)	0.39 (0.21–0.71)	908 (63.7)
	≥11	75.8 (63.0–85.4)	46.3 (43.6–49.0)	6.0 (4.5–8.0)	97.7 (96.1–98.7)	1.41 (1.22–1.64)	0.52 (0.34–0.82)	780 (54.7)
	≥12	67.7 (54.5–78.7)	55.9 (53.2–58.5)	6.5 (4.8–8.8)	97.4 (96.0–98.4)	1.53 (1.28–1.84)	0.58 (0.40–0.83)	644 (45.2)
	**≥13**	**64.5 (51.3–76.0)**	**64.2 (61.6–66.8)**	**7.6 (5.5–10.3)**	**97.6 (96.3–98.4)**	**1.80 (1.48–2.20)**	**0.55 (0.39–0.77)**	**528 (37.0)**
	≥14	54.8 (41.8–67.3)	73.5 (71.0–75.8)	8.6 (6.1–11.9)	97.3 (96.0–98.2)	2.07 (1.62–2.63)	0.61 (0.47–0.81)	396 (27.8)
	≥15	41.9 (29.8–55.1)	79.1 (76.8–81.2)	8.4 (5.6–12.2)	96.8 (95.5–97.7)	2.01 (1.47–2.74)	0.73 (0.59–0.91)	311 (21.8)
	≥16	33.9 (22.7–47.1)	85.6 (83.6–87.4)	9.7 (6.2–14.6)	96.6 (95.4–97.5)	2.36 (1.63–3.42)	0.77 (0.65–0.92)	217 (15.2)
OGTT and HbA1c (N: 1,426 participants)								
	≥9	96.2 (90.0–98.8)	29.8 (27.4–32.4)	9.8 (8.1–11.9)	99.0 (97.3–99.7)	1.37 (1.30–1.44)	0.13 (0.05–0.34)	1,028 (72.1)
	≥10	85.7 (77.2–91.5)	38.1 (35.5–40.8)	9.9 (8.1–12.1)	97.1 (95.2–98.3)	1.38 (1.27–1.51)	0.38 (0.23–0.60)	908 (63.7)
	≥11	79.1 (69.8–86.1)	47.2 (44.5–50.0)	10.6 (8.6–13.1)	96.6 (94.8–97.8)	1.50 (1.34–1.67)	0.44 (0.30–0.65)	780 (54.7)
	≥12	70.5 (60.7–78.8)	56.9 (54.1–59.5)	11.5 (9.2–14.3)	96.0 (94.3–97.3)	1.63 (1.42–1.88)	0.52 (0.39–0.70)	644 (45.2)
	**≥13**	**63.8 (53.8–72.8)**	**65.1 (62.4–67.7)**	**12.7 (10.0–15.9)**	**95.8 (94.2–97.0)**	**1.83 (1.56–2.15)**	**0.56 (0.43–0.72)**	**528 (37.0)**
	≥14	54.3 (44.3–64.0)	74.3 (71.9–76.7)	14.4 (11.2–18.3)	95.3 (93.8–96.5)	2.12 (1.74–2.58)	0.61 (0.50–0.76)	396 (27.8)
	≥15	46.7 (37.0–56.6)	80.2 (77.9–82.3)	15.8 (12.0–20.4)	95.0 (93.5–96.2)	2.35 (1.87–2.97)	0.67 (0.56–0.80)	311 (21.8)
	≥16	38.1 (28.9–48.1)	86.6 (84.6–88.4)	18.4 (13.6–24.4)	94.6 (93.2–95.8)	2.84 (2.15–3.76)	0.71 (0.61–0.83)	217 (15.2)

On the basis of OGTT criteria alone, the performance of FINDRISC changed: the best cut-off point was ≥15 (sensitivity 45% and specificity 79.5%), whereas for HbA1c criteria alone, the best cut-off point was ≥14 (sensitivity 64.4% and specificity 73.4%). Finally, for FPG criteria alone, the best cut-off point, which was ≥13 (sensitivity 64.5% and specificity 64.2%).

For dysglycaemia, a cut-off point of ≥12 in the FINDRISC score offered the best balance between true-positive and false-positive rates when HbA1c criteria alone or OGTT and HbA1c criteria were used. However, the best cut-off point was ≥13 based on OGTT criteria alone and ≥11 for FPG criteria alone (Table 4).

**Table 4:** Characteristics of the FINDRISC questionnaire using different cut-off values for dysglycaemia.

GOLD STANDARD	FINDRISC Cut-off	Sensitivity (%) (95%CI)	Specificity (%) (95%CI)	PPV (%) (95%CI)	NPV (%) (95%CI)	LR+ (95%CI)	LR- (95%CI)	N (%)
HbA1c (N: 1,425 participants)								
	≥9	78.3 (75.1–81.2)	34.4 (30.9–38.1)	56.0 (52.9–59.1)	59.7 (54.7–64.5)	1.19 (1.12–1.27)	0.63 (0.53–0.75)	1,028 (72.1)
	≥10	70.7 (67.2–73.9)	43.7 (40.0–47.5)	57.3 (54.0–60.5)	58.2 (53.8–62.5)	1.25 (1.16–1.36)	0.67 (0.58–0.77)	908 (63.7)
	≥11	63.3 (59.7–66.8)	54.4 (50.6–58.2)	59.7 (56.2–63.2)	58.1 (54.2–62.0)	1.39 (1.26–1.53)	0.67 (0.60–0.76)	780 (54.7)
	**≥12**	**54.1 (50.4–57.7)**	**64.3 (60.6–67.9)**	**61.8 (57.9–65.6)**	**56.7 (53.2–60.2)**	**1.51 (1.34–1.71)**	**0.71 (0.66–0.79)**	**644 (45.2)**
	≥13	45.0 (41.4–48.7)	71.4 (67.9–74.7)	62.7 (58.4–66.8)	54.9 (51.5–58.1)	1.57 (1.36–1.81)	0.77 (0.71–0.84)	528 (37.0)
	≥14	35.6 (32.2–39.2)	80.6 (77.4–83.4)	66.2 (61.2–70.8)	53.9 (50.8–57.0)	1.83 (1.53–2.19)	0.80 (0.75–0.85)	396 (27.8)
	≥15	28.5 (25.3–32.0)	85.3 (82.4–87.9)	67.5 (62.0–72.6)	52.8 (49.8–55.7)	1.95 (1.57–2.41)	0.84 (0.79–0.88)	311 (21.8)
	≥16	21.1 (18.2–24.2)	91.0 (88.6–93.0)	71.4 (64.9–77.2)	51.9 (49.0–54.8)	2.34 (1.78–3.08)	0.87 (0.83–0.91)	217 (15.2)
OGTT (N: 1,408 participants)								
	≥9	85.1 (80.9–88.6)	32.4 (29.6–35.4)	29.4 (26.7–32.4)	86.8 (83.0–89.9)	1.26 (1.19–1.34)	0.46 (0.35–0.60)	1,028 (72.1)
	≥10	78.9 (74.1–82.9)	41.4 (38.4–44.4)	30.8 (27.8–34.0)	85.6 (82.1–88.4)	1.35 (1.25–1.45)	0.51 (0.41–0.63)	908 (63.7)
	≥11	71.7 (66.6–76.3)	51.0 (47.9–54.0)	32.6 (29.3–36.1)	84.5 (81.4–87.2)	1.46 (1.34–1.60)	0.56 (0.47–0.66)	780 (54.7)
	≥12	62.3 (57.0–67.3)	60.5 (57.5–63.4)	34.3 (30.6–38.1)	82.9 (80.0–85.5)	1.58 (1.41–1.76)	0.62 (0.54–0.72)	644 (45.2)
	**≥13**	**54.3 (48.9–59.6)**	**68.7 (65.8–71.5)**	**36.5 (32.4–40.8)**	**82.0 (79.2–84.4)**	**1.74 (1.52–1.98)**	**0.67 (0.59–0.75)**	**528 (37.0)**
	≥14	44.6 (39.3–50.0)	77.8 (75.1–80.2)	39.9 (35.0–45.0)	80.9 (78.3–83.3)	2.01 (1.71–2.36)	0.71 (0.65–0.79)	396 (27.8)
	≥15	37.4 (32.3–42.8)	83.3 (80.9–85.4)	42.5 (37.0–48.3)	80.1 (77.6–82.4)	2.24 (1.85–2.71)	0.75 (0.69–0.82)	311 (21.8)
	≥16	28.3 (23.7–33.4)	89.0 (87.0–90.8)	46.1 (39.3–53.0)	79.0 (76.5–81.2)	2.58 (2.03–3.28)	0.81 (0.75–0.86)	217 (15.2)
FPG (N: 1,426 participants)								
	≥9	79.1 (75.8–82.0)	34.5 (31.1–38.1)	53.2 (50.1–56.3)	63.6 (58.6–68.3)	1.21 (1.13–1.29)	0.61 (0.51–0.72)	1,028 (72.1)
	≥10	70.7 (67.1–74.0)	42.9 (39.3–46.6)	53.9 (50.6–57.1)	60.8 (56.4–65.0)	1.24 (1.14–1.34)	0.68 (0.59–0.79)	908 (63.7)
	**≥11**	**62.0 (58.3–65.6)**	**52.2 (48.5–55.8)**	**55.0 (51.4–58.5)**	**59.3 (55.4–63.1)**	**1.30 (1.18–1.43)**	**0.73 (0.65–0.82)**	**780 (54.7)**
	≥12	51.8 (47.9–55.5)	61.0 (57.4–64.6)	55.6 (51.7–59.5)	57.3 (53.7–60.8)	1.33 (1.18–1.49)	0.79 (0.72–0.87)	644 (45.2)
	≥13	43.1 (39.4–46.9)	68.7 (65.2–72.0)	56.4 (52.1–59.4)	56.1 (52.8–59.4)	1.37 (1.20–1.58)	0.83 (0.76–0.90)	528 (37.0)
	≥14	34.0 (30.5–37.6)	78.1 (74.9–81.0)	59.3 (54.3–64.2)	55.6 (52.5–58.7)	1.55 (1.30–1.84)	0.85 (0.79–0.90)	396 (27.8)
	≥15	27.6 (24.3–31.1)	83.7 (80.7–86.2)	61.4 (55.7–66.8)	55.1 (52.1–58.0)	1.69 (1.38–2.07)	0.87 (0.82–0.92)	311 (21.8)
	≥16	21.0 (18.0–24.2)	90.2 (87.8–92.2)	66.8 (60.1–73.0)	54.8 (51.9–57.6)	2.14 (1.64–2.78)	0.88 (0.84–0.92)	217 (15.2)
OGTT and HbA1c (N: 1,426 participants)								
	≥9	78.0 (75.1–80.7)	36.7 (32.8–40.8)	64.6 (61.6–67.5)	53.0 (48.0–58.0)	1.23 (1.15–1.32)	0.60 (0.51–0.71)	1,028 (72.1)
	≥10	70.4 (67.2–73.4)	46.3 (42.1–50.4)	66.0 (62.8–69.0)	51.4 (47.0–55.7)	1.31 (1.20–1.43)	0.64 (0.56–0.73)	908 (63.7)
	≥11	62.4 (59.0–65.7)	56.7 (52.5–60.8)	68.1 (64.7–71.3)	50.5 (46.5–54.4)	1.44 (1.29–1.60)	0.66 (0.59–0.74)	780 (54.7)
	**≥12**	**53.0 (49.6–56.4)**	**66.4 (62.4–70.3)**	**70.0 (66.3–73.5)**	**48.9 (45.3–52.4)**	**1.58 (1.38–1.80)**	**0.71 (0.65–0.78)**	**644 (45.2)**
	≥13	44.5 (41.2–48.0)	74.1 (70.3–77.6)	71.8 (67.7–75.5)	47.4 (44.1–50.8)	1.72 (1.47–2.01)	0.75 (0.69–0.81)	528 (37.0)
	≥14	34.6 (31.4–37.9)	82.3 (78.8–85.3)	74.2 (69.6–78.4)	45.9 (42.9–49.0)	1.95 (1.60–2.38)	0.80 (0.75–0.85)	396 (27.8)
	≥15	27.9 (24.9–31.0)	87.1 (84.1–89.7)	76.2 (71.0–80.8)	44.9 (42.0–47.9)	2.16 (1.70–2.75)	0.83 (0.79–0.87)	311 (21.8)
	≥16	20.5 (17.8–23.4)	92.5 (90.0–94.5)	80.2 (74.1–85.2)	44.0 (41.2–46.9)	2.73 (1.99–3.75)	0.86 (0.83–0.90)	217 (15.2)

**Fig 1:**
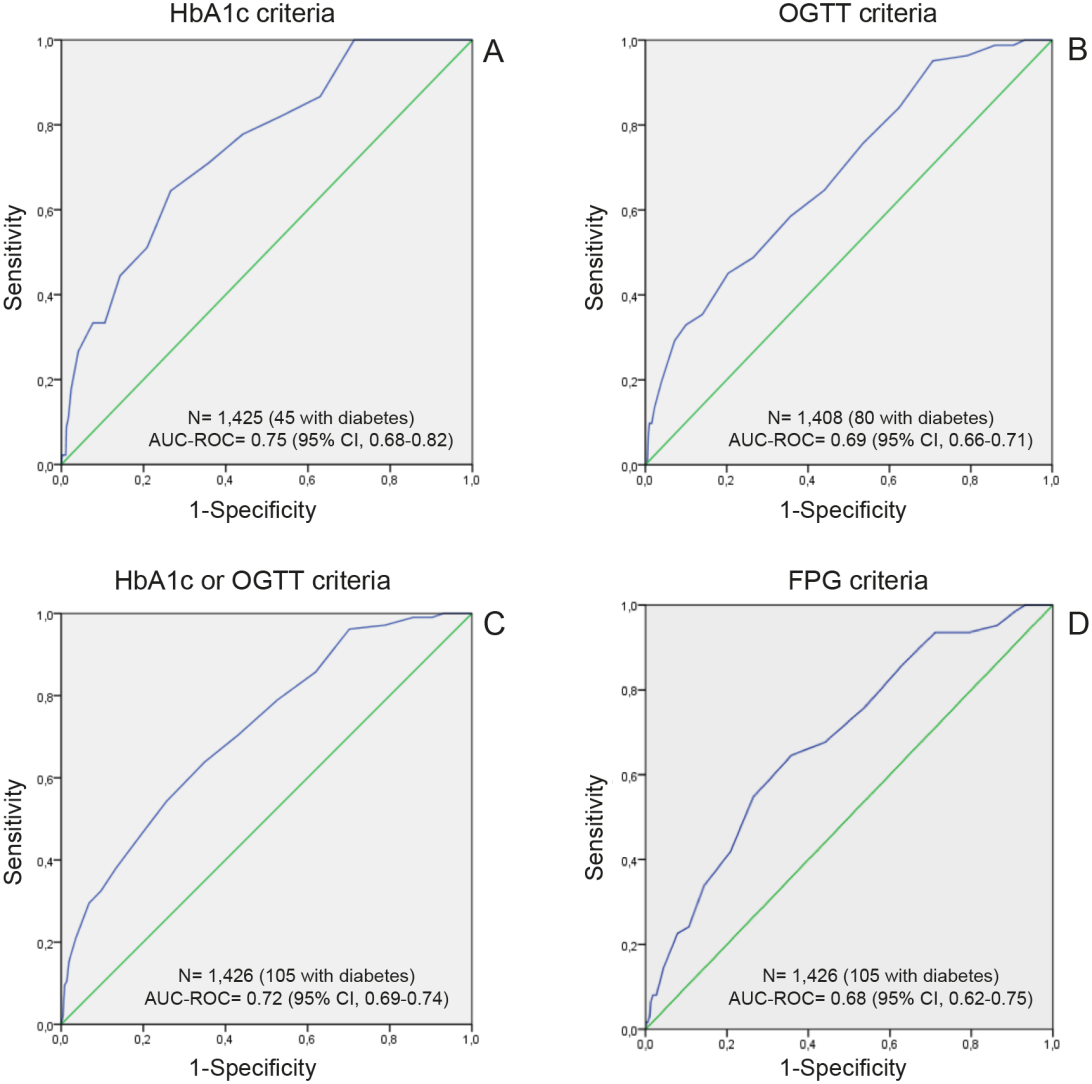
Receiver operating characteristic curves in patients with newly diagnosed diabetes based on three diagnostic criteria: HbA1c (A), OGTT (B), HbA1c and OGTT (C), and FPG (D).

Finally, the area under the ROC curve (AUC) for UT2DM based on HbA1c criteria alone (Fig 1A) was 0.75 (95% CI, 0.68–0.82), 0.69 (95% CI, 0.66–0.71) based on OGTT criteria alone (Fig 1b), 0.72 (95% CI, 0.69–0.74) based on OGTT or HbA1c criteria (Fig 1c), and 0.68 (95% CI, 0.62–0.75) for FPG criteria (Fig 1d).

The AUC for dysglycaemia was 0.63 (95% CI, 0.61–0.66) based on OGTT or HbA1c criteria, 0.67 (95% CI, 0.63–0.70) for OGTT alone, 0.62 (95% CI, 0.59–0.65) for HbA1c alone, and 0.60 (95% CI, 0.57–0.63) for FPG.

In the MADRISC, the variables most strongly associated with UDM in the univariate analysis were BMI, history of blood glucose disorders, and use of blood pressure medication. Multiple logistic regression analysis revealed the variables most strongly associated with UDM to be BMI ≥30 kg/m^2^ (β coefficient = 2.006; OR = 7.43; p<0.01), history of blood glucose disorders (β coefficient = 1.34; OR = 3.83; p<0.01), BMI between 25 and 30 kg/m^2^ (β coefficient = 1.118; OR = 3.06; p = 0.013), and, use of blood pressure medication (β coefficient = 0.833; OR = 2.30; p<0.01). The scores were assigned as follows: BMI <25 kg/m^2^, 0 points; BMI 25–30 kg/m^2^, 11 points; BMI ≥30 kg/m^2^, 20 points; positive history of blood glucose disorders, 13 points; negative history of blood glucose disorders, 0 points; use of blood pressure medication, 8 points; no use of blood pressure medication, 0 points. Based on OGTT or HbA1c criteria, the best cut-off point was ≥13 (sensitivity 84.8% and specificity 54.6%) (Table 5) and the AUC was 0.76 (95% CI, 0.72–0.81), both of which are considered discriminatory. The Hosmer-Lemeshow test showed that the expected and observed UDM rates were very similar and that the model was well calibrated (chi-square = 4.52, df = 5, p = 0.477).

**Table 5:** Characteristics of the simplified FINDRISC (MADRISC) questionnaire using different cut-off values for undiagnosed diabetes mellitus.

GOLD STANDARD	MADRISC Cut-off	Sensitivity (%) (95%CI)	Specificity (%) (95%CI)	PPV (%) (95%CI)	NPV (%) (95%CI)	LR+(95%CI)	LR-(95%CI)
OGTT and/or HbA1c (N: 1,426 participants)							
	≥8	99.1 (94.0–99.9)	19.6 (17.5–21.9)	8.9 (7.4–10.7)	99.6 (97.5–99.9)	1.23 (1.19–1.27)	0.05 (0.01–0.34)
	≥11	96.2 (89.9–98.8)	24.8 (22.5–27.2)	9.2 (7.6–11.1)	98.8 (96.7–99.6)	1.28 (1.22–1.34)	0.15 (0.06–0.40)
	**≥13**	**84.8 (76.1–90.8)**	**54.6 (51.9–57.3)**	**12.9 (10.6–15.7)**	**97.8 (96.4–98.7)**	**1.87 (1.69–2.06)**	**0.28 (0.18–0.44)**
	≥19	82.9 (74.0–89.3)	55.7 (53.0–58.4)	13.0 (10.6–15.8)	97.6 (96.2–98.5)	1.87 (1.68–2.08)	0.31 (0.20–0.47)
	≥20	69.5 (59.7–77.9)	67.7 (65.1–70.2)	14.6 (11.7–18.1)	96.5 (95.1–97.6)	2.15 (1.85–2.50)	0.45 (0.34–0.60)
	≥21	51.4 (41.5–61.2)	81.8 (79.5–83.8)	18.3 (14.2–23.3)	95.5 (94.1–96.6)	2.82 (2.27–3.51)	0.59 (0.49–0.72)
	≥24	51.4 (41.5–61.2)	82.3 (80.1–84.3)	18.8 (14.5–23.9)	95.5 (94.1–96.6)	2.90 (2.33–3.61)	0.59 (0.48–0.72)
	≥28	31.5 (27.3–35.9)	92.6 (83.7–87.5)	20.4 (15.7–26.0)	95.5 (94.1–96.6)	3.22 (2.56–4.06)	0.60 (0.49–0.72)
	≥32	24.8 (17.1–34.3)	96.1 (94.8–97.0)	33.3 (23.3–45.0)	94.1 (92.7–95.3)	6.29 (4.11–9.64)	0.78 (0.70–0.87)
	≥33	18.1 (11.5–27.1)	97.2 (96.1–98.0)	33.9 (22.2–47.9)	93.7 (92.3–94.9)	6.46 (3.86–10.83)	0.84 (0.77–0.92)
	≥41	12.4 (07.0–20.6)	98.4 (97.5–99.0)	38.2 (22.7–56.4)	93.4 (91.9–94.6)	7.79 (4.02–15.11)	0.89 (0.83–0.96)

## Discussion

Our study showed that the prevalence of dysglycaemia based on HbA1c or OGTT in the population of northen Madrid aged 45–75 years was high: 59.7% for dysglycaemia and 7.4% for previously undiagnosed or newly diagnosed T2DM.

Based on OGTT criteria, the prevalence of UT2DM (5.6%) is similar to that reported in the study by Soriguer *et al*. [21], which is the most representative study on UT2DM in Spain. The authors designed a population-based, cross-sectional study with cluster sampling and found the prevalence of UDM to be 6%. However, we found the prevalence of dysglycaemia based on OGTT criteria to be lower (24.9% vs. 30%).

The prevalence of UDM is more likely to be lower when the diagnosis is based on HbA1c than when it is based on OGTT, as noted in previous studies [22]. Our data confirmed this finding. Consequently, although it may seem preferable to perform OGTT, recent reports have suggested that individuals in the early stages of DM meet the HbA1c criteria and display a more unfavorable cardiovascular risk profile than those who fulfill only the OGTT criteria [23]. In addition, previous results reported by our group showed that prediabetes diagnosed based on HbA1c criteria was more likely to predict the presence of carotid plaques than when diagnosed based on OGTT [24].

The FINDRISC was originally developed in a prospective cohort to identify people at high risk of developing T2DM [12], and the cross-sectional studies that have analyzed the performance of this score as a screening tool for detection of UDM [16] show that the optimal cut-off points vary widely, from 9 [12] to 15 [25]. This variability could be due to the need to assess the instrument in its target population [26].

Based on OGTT or HbA1c criteria, we found that ≥13 was the optimal cut-off point for identifying individuals with UT2DM. Sensitivity was 63.8%, specificity 65.1%, and the negative predictive value 95.8%, all of which were similar to the values in the original FINDRISC cross-sectional study [27]. Our cut-off point is higher than those reported in cross-sectional studies from Southern Europe [13,28], Germany [29–31], the United Kingdom [32], Finland [12], and the Philippines [33]. However, consistent with the original cut-off point in the Lindström study [12], several of these European studies [13,28–30,32] have used ≥9 as their best cut-off point, without applying the peaks of the ROC curve, where the sum of sensitivity and specificity are at a maximum.

It seems that the gold standard for determining the performance of FINDRISC may play a key role. For most studies, the OGTT and/or FPG are considered the gold standard, although Zhang *et al*. [34] proposed using FPG, OGTT, or HbA1c criteria. Indeed, the Zhang study showed a higher sensitivity (75% in men, and 72% in women) and AUC (0.75) than other studies that used OGTT and/or FPG criteria. Only Li *et al*. [31] and Lindstrom *et al*. [12], whose studies were based on OGTT and/or FPG criteria, showed a higher AUC than Zhang *et al*. [34].

In Spain, Costa *et al*. [14] carried out a study to detect UT2DM and dysglycaemia using only FPG, OGTT, and HbA1c and not including combinations of these criteria. Based on the AUC, the authors concluded that OGTT and FPG have better overall discriminatory power than HbA1c.

In contrast, considering our data with HbA1c as the gold standard, the AUC of FINDRISC was higher than that found by Costa et al. [14] for detection of both UT2DM and dysglycaemia.

Regardless of the criteria used, the ROC curves in the study by Costa et al. [14] indicated that ≥14 was the best cut-off. In the present study, with a similar population, the best cut-off value for detection of UDM was 1 point lower. These small differences can be explained by the sampling method, based in the active public-health program, DE-PLAN (Diabetes in Europe-Prevention using Lifestyle, Physical Activity and Nutritional intervention), used in the Costa study.

The best cut-off point for identifying dysglycaemia (prediabetes + newly diagnosed T2DM) with FINDRISC was ≥12 based on the OGTT or HbA1c criteria and ≥13 using OGTT criteria alone. Once again, these results reveal differences in the design of the studies and between populations in Southern Europe [14,25], many of whom have higher cut-off values. However, our data revealed that the power of FINDRISC to detect dysglycaemia is poor.

The MADRISC questionnaire showed good discriminatory power, with a ROC-AUC of 0.76 (95% CI, 0.72–0.81). The best cut-off was ≥13, with a sensitivity of 84.8% and a specificity of 54.6%. Furthermore, the Hosmer-Lemeshow test showed that the MADRISC questionnaire was well calibrated (chi-square = 4.52, df = 5, p = 0.477).

A simplified FINDRISC questionnaire was applied in Germany by Li et al. [31]. The version included age, BMI, and history of high blood glucose, and the values for the AUC (0.86), sensitivity (79%), and specificity (80%) were excellent. Bergmann [30] also developed a simplified six-item FINDRISC questionnaire based on the following variables: age, waist circumference, BMI, blood pressure medication, history of high blood glucose, physical activity, daily consumption of fruits, berries, and vegetables. The AUC was 0.74, sensitivity 70%, and specificity 63%.

The advantage of the MADRISC risk score is its simplicity, since it includes only three variables (BMI, history of antihypertensive drug treatment, and history of blood glucose disorders), which are easily collected in clinical practice. Therefore, pre-screening values will be positive in patients with a BMI ≥30 kg/m^2^ (20 points) or with a history of blood glucose disorders (13 points) and in patients with a BMI between 25 and 29 kg/m^2^ (11 points) plus a history of antihypertensive drug treatment (8 points; total = 11+8 = 19 points) or plus a history of blood glucose disorders (13 points; total = 11+13 = 24 points).

The variables included in MADRISC were well accepted in a sample of 1,081 employees at Karolinska University Hospital who did not complete the entire FINDRISC score. The complete response ranged from 82% for BMI to 92% for history of high blood glucose [35]. As mentioned above, time is a limited resource in the primary care setting, and the use of a simplified FINDRISC score could improve the efficiency of the tool.

Althought the reduced model helps to facilitate pre-screening as a suitable strategy for identifying people at risk of DM, its effectiviness will depend on successful uptake of screening [36]. Indeed, we think that a short questionnaire on risk of DM that does not include variables that require measurement (e.g., waist circumference) is an excellent strategy for improving the identification of UDM.

The original FINDRISC and other diabetes screening tools such as the QD Score and Cambridge Risk Score are considered useful screening strategies [37]. However, as these scores have a limited positive predictive value, they are more useful for specific population subgroups with a higher prevalence of unknown diabetes (e.g., elderly patients or patients with a family history of diabetes mellitus) than for the general population. In our opinion, this limitation is the same as that of the simplified MADRISC. Therefore, the scores can also be applied as a preliminary tool, with a recommended second phase involving a blood test.

Our study has several limitations. First, since the sample was recruited from two districts in the northern metropolitan area of Madrid, the results may not be applicable to the general population of southern Madrid, which is characterized by a lower educational and socioeconomic level and, consequently, higher risk of dysglycaemia [38]. However, our results are similar to those found in Greece [25], where the socioeconomic level is lower than in Spain; therefore, we do not believe that a potential sampling bias seriously affects our findings. Second, the diagnosis of DM and dysglycaemia was not confirmed by repeat testing on a separate day. This limitation is shared by other studies in the field. However, a certain degree of imperfect gold standard bias may have led us to overestimate sensitivity and specificity [39]. On the other hand, the strengths of the study were that the age distribution of the participants was wide (45–75 years) and the study was population-based. Therefore, we believe that the data are generalizable to other metropolitan populations.

## Conclusions

Our data call into question the original FINDRISC cut-off point used to identify UT2DM and dysglycaemia, at least in a representative Spanish population living in a metropolitan area. Therefore, a FINDRISC value ≥12 is the most suitable value for identifying patients with dysglycaemia on the basis of HbA1c criteria alone or the combination of HbA1c plus OGTT criteria. A cut-off point ≥13 proved optimal for a diagnosis of T2DM based on OGTT or HbA1c. Therefore, scores traditionally considered moderate risk for diabetes (12–14 points) proved to be more effective than higher cut-offs for diagnosing diabetes and dysglycaemia in our study.

The FINDRISC performed well as a screening tool for the cross-sectional detection of dysglycaemia and UT2DM. In the screening of UT2DM, MADRISC performed as well as FINDRISC. Considering the primary care setting and time resource constraints, the MADRISC is preferred.

## Supporting Information

S1 Table Differences between participants and non-participants at recruitment phase. (DOCX)

## Abbreviations

BMI: Body mass index
DM: Diabetes mellitus
FPG: Fasting plasma glucose
FINDRISC: Finnish Diabetes Risk Score
OGTT: Oral glucose tolerance test
SPREDIA-2: Screening PRE-diabetes and type 2 DIAbetes
T2DM: Type 2 diabetes mellitus
UDM: Undiagnosed diabetes mellitus
UT2DM: Undiagnosed type 2 diabetes mellitus
WHO: World Health Organization
