# Supplementary material for: Performance of the Finnish Diabetes Risk Score and a Simplified Finnish Diabetes Risk Score in a Community-Based, Cross-Sectional Programme for Screening of Undiagnosed Type 2 Diabetes Mellitus and Dysglycaemia in Madrid, Spain: The SPREDIA-2 Study
Source: PLoS One. 2016 Jul 21;11(7):e0158489. doi: 10.1371/journal.pone.0158489 (PMC4956208; doi:10.1371/journal.pone.0158489)
Supplement: S1 Table — (DOCX) [file pone.0158489.s001.docx]

**Table S1. Differences between participants and non-participants at recruitment phase.**

|  |  |  |  |  |
| --- | --- | --- | --- | --- |
|  | **Participants**  **(N=1,592)** | **Non participants**  **(N=1,001)** | | **p-value** |
| **Female, gender** % (n) | 58.3 (928) | 56.5 (566) | | 0.367 |
| **Age**, *mean (SD)* | 61.7 (6) | 61.3 (6.2) | | 0.103 |
| **Family history of DM**, % (n) | 30.6 (487) | 25.7 (257) | | < 0.01 |
| **Hypertension,** % (n) | 34.9 (556) | 22.5 (225) | | <0.01 |
| **Dyslipidemia,** % (n) | 42.9 (683) | 28.0 (280) | | <0.01 |
| **BMI Kg/m^2^,** *mean (SD)* | 28.2 (4.7) | 27.9 (4.1)* | | 0.090 |

*Calculated from Weight and Height self-reported
